# The association between body mass index and live birth and maternal and perinatal outcomes after *in-vitro* fertilization: a national cohort study

**DOI:** 10.3389/fendo.2023.1239702

**Published:** 2023-09-11

**Authors:** Linda Kluge, Karin Källén, Ann Thurin-Kjellberg, Ulla-Britt Wennerholm, Christina Bergh

**Affiliations:** ^1^ Department of Obstetrics and Gynaecology, Institute of Clinical Science, Sahlgrenska Academy, Gothenburg University, Gothenburg, Sweden; ^2^ Reproductive Medicine, Sahlgrenska University Hospital, Gothenburg, Sweden; ^3^ Department of Reproduction Epidemiology, Tornblad Institute, Institute of Clinical Science, Lund University, Lund, Sweden; ^4^ Department of Obstetrics and Gynaecology, Region Västra Götaland, Sahlgrenska University Hospital, Gothenburg, Sweden

**Keywords:** obesity, *in vitro* fertilization, live birth, maternal and perinatal outcomes, population registers

## Abstract

**Objective:**

To investigate the association between female body mass index (BMI) and live birth rates and maternal and perinatal outcomes after *in-vitro* fertilization (IVF).

**Methods:**

We performed a national, population-based cohort study including women undergoing IVF between 2002 and 2020. The cohort included 126,620 fresh cycles and subsequent frozen embryo transfers between 2007 and 2019 (subpopulation 1) and 58,187 singleton deliveries between 2002 and 2020 (subpopulation 2). Exposure was female BMI (kg/m^2^) categorized according to the World Health Organization as underweight (<18.5), normal weight (18.5–24.9, reference), overweight (25.0–29.9), class I obesity (30.0–34.9), class II obesity (35.0–39.9), and class III obesity (≥40.0). The primary outcome in subpopulation 1 was cumulative live birth per started fresh IVF cycle, including fresh and subsequent frozen embryo transfers. Primary outcomes in subpopulation 2 were hypertensive disorders of pregnancy and preterm birth at less than 37 weeks. Risk ratios (RRs) with 95% confidence intervals (CIs) for the association between BMI class and outcomes were calculated using generalized linear models after adjustment for relevant confounders.

**Results:**

The cumulative live birth rate decreased significantly with increasing BMI from 32.6% in normal-weight women to 29.4% in overweight women, 27.0% in women in obesity class I, 21.8% in women in obesity class II, and 7.6% in women in obesity class III. The risk of hypertensive disorders of pregnancy increased significantly and progressively with increasing BMI, from 4.6% in normal-weight women to 7.8% in overweight women and 12.5%, 17.9%, and 20.3% in women in obesity classes I, II, and III. The risk of preterm birth followed a similar pattern, from 6.3% in normal-weight women to 7.5% in overweight women and 8.9%, 9.9%, and 15.3% in women in obesity classes I, II, and III. The risks of other perinatal complications, such as perinatal death, showed an even more pronounced increase.

**Conclusion:**

Using a large and complete national cohort of women undergoing IVF, we demonstrate a dose-dependent decrease in live birth rate and a substantial increase in maternal and perinatal complications with increasing BMI. Strategies to improve this situation are warranted.

## Introduction

Obesity is an increasing global health problem, and according to the World Health Organization (WHO), obesity has become three times more common all over the world in 2019 than in 1975 (1). In Sweden, in 2021, 28.4% of women in early pregnancy were overweight and 16.8% were obese (2). In the United States in 2020, the prevalence rates of women with self-reported pre-pregnancy overweight and obesity were 27.2% and 30.1% (3). Obesity has an impact on many health issues including female fertility and obstetric outcomes. Several studies have shown a negative association between high body mass index (BMI) and live birth after *in-vitro* fertilization (IVF) (4–8).

In spontaneously conceived pregnancies, overweight and obese women have an increased risk of adverse outcomes, such as a higher miscarriage rate and increased risk of gestational diabetes and hypertensive disorders of pregnancy, than normal-weight women (9–12). Children born to overweight and obese women have a higher incidence of adverse outcomes such as birth defects, stillbirth, and neonatal and infant mortality than children born to normal-weight women (11, 13–16). The association between BMI and obstetric and perinatal outcomes after IVF is less explored. However, studies have shown that compared with normal-weight women, overweight and obese women undergoing IVF have an increased risk of preterm birth (less than 37 weeks) and delivery of large-for-gestational-age infants (17–19).

The objective of this study was to investigate the association between BMI and live birth rates in a complete, national cohort of women undergoing IVF. Furthermore, maternal and perinatal outcomes were assessed. The results may serve as a guide to both patients and IVF units.

## Materials and methods

### Setting

This retrospective nationwide population-based register study was performed in Sweden where up to three publicly funded IVF treatments are offered to women below 40 years of age with no children in their present relationship. In addition, IVF is performed at several private IVF clinics.

### Study population

Women undergoing IVF between 2002 and 2020 were included. Treatments with donated oocytes and preimplantation genetic testing were excluded. Furthermore, cycles with own oocyte donation for social freezing and for fertility preservation were excluded since these cycles were not planned for embryo transfer in the near future. In the analysis of live birth rate, and after these exclusions, we retrieved information from 150,847 started fresh IVF/ICSI (intracytoplasmic sperm injection) cycles (*n* = 66,568 women) performed in all Swedish IVF clinics between 1 January 2007 and 31 December 2019 and all subsequently performed frozen embryo transfers within 1 year (subpopulation 1). We excluded 24,227 (16.1%) started fresh cycles because information on BMI was missing.

In the analysis of maternal and perinatal outcomes, we retrieved information on all fresh and frozen embryo transfers performed in all Swedish IVF clinics between 1 January 2002 and 31 December 2020 that resulted in singleton deliveries, with a total of 60,095 deliveries (*n* = 50,651 women) (subpopulation 2). Stillbirths were included. Due to missing BMI, 1,905 (3.2%) of the deliveries were excluded. Women treated with donated eggs and preimplantation genetic testing were excluded.

### Data sources

The Swedish personal identification number assigned to each permanent resident enables linkage between several health data registers, quality registers, and population-based registers in Sweden. In 2007, the Swedish National Quality Register of Assisted Reproduction (Q-IVF) (20) was established. The register has almost 100% coverage (www.qivf.se).

For subpopulation 1, we retrieved all data from the Q-IVF, except infertility diagnoses, educational level, and country of birth. Data on infertility diagnoses were retrieved from the Swedish National Patient Registry (NPR) and data on educational level and country of birth from Statistics Sweden (SCB) (Statistics Sweden, 2022). Data from the Q-IVF were cross-linked with the NPR and SCB.

For subpopulation 2, IVF data were identified in two registers: a research data set stored at the Swedish Medical Birth Register (MBR) here called MBR-IVF for treatments performed between 2002 and 2006 and from the Q-IVF for treatments performed between 2007 and 2020. Data from the MBR-IVF and Q-IVF were cross-linked with those in the MBR and the Swedish Neonatal Quality Register (SNQ) for maternal and perinatal outcomes and in the NPR for infertility diagnoses, the Swedish Cause of Death Register (CDR) for data on neonatal death, and the SCB for data on maternal educational level and country of birth. The MBR, CDR, and NPR are mandatory registers hosted by the Swedish National Board of Health and Welfare (21). The MBR was established in 1973 and covers data on 98% of all deliveries in Sweden (22). All causes of death are registered in the CDR, established in 1952, and have full coverage (23). The NPR was launched in 1987, the register covers all in-patient care, and since 2001, the register also covers outpatient specialized care and was validated in 2011 (24). The SNQ was established in 2001 and includes information on infants admitted to neonatal intensive care units (25). The cross linkage of data was performed by the Swedish National Board of Health and Welfare. The data received are pseudonymized, meaning that researchers got a data set with serial numbers instead of personal identification numbers.

### Main exposure

BMI was the main exposure. BMI was assessed from measured weight and self-reported height and calculated as the woman’s weight in kilograms divided by the square of height in meters. For subpopulation 1, data on BMI in relation to each started fresh IVF cycle were retrieved from the Q-IVF, measured at oocyte retrieval. For subpopulation 2, data on BMI were retrieved from the MBR from the first prenatal visit or if missing in the MBR from the Q-IVF. BMI was categorized according to the WHO classifications as underweight (<18.5 kg/m²), normal weight (18.5–24.9 kg/m²), overweight (25.0–29.9 kg/m²), class I obesity (30.0–34.9 kg/m²), class II obesity (35.0–39.9 kg/m²), and class III obesity (≥40.0 kg/m²).

### Outcomes

The primary outcome in subpopulation 1 was cumulative live birth, defined as the number of deliveries with at least one live born child (singleton or multiple) per started fresh IVF cycle, including one fresh and/or all frozen embryo transfers within 1 year, until one delivery with a live birth or until all embryos were used, whichever occurred first (26). Thus, also started cycles canceled before oocyte retrieval or before any embryo transfer were included in the denominator. A started fresh IVF cycle was defined as a fresh cycle where at least one dose of gonadotrophins was administered. Secondary outcomes were live birth per started fresh IVF cycle, live birth per fresh embryo transfer and per first embryo transfer (fresh or frozen), miscarriage among clinical pregnancies after first embryo transfer, and multifetal pregnancy rate among deliveries after the first embryo transfer. Cumulative live birth rate and live birth after first embryo transfer are today considered more accurate ways of measuring success after IVF than live birth after fresh embryo transfer due to the change in embryo transfer policy, which has resulted in a dramatic increase of frozen cycles all over the world (27). In a substantial number of cycles, no fresh embryo transfer takes place, mainly due to risk of ovarian hyperstimulation syndrome (OHSS), and all embryos are frozen for a later embryo transfer. A clinical pregnancy was defined as ultrasonographic visualization of one or more gestational sacs (26).

In subpopulation 2, the primary outcomes were hypertensive disorders of pregnancy, classified as gestational hypertension [International Classification of Diseases, 10th revision (ICD-10) (28) O13], preeclampsia (O14), or eclampsia (O15), and preterm birth defined as birth after less than 37 gestational weeks and 0 days (37 + 0). The secondary outcomes were emergency cesarean section (O82.1), stillbirth or neonatal death (P95), Apgar score less than 7 at 5 min, birth trauma (P10–P15), admission to a neonatal intensive care unit for more than 4 days, and major birth defects. In Sweden, until July 2008, stillbirths were defined as deliveries at ≥28 + 0 gestational weeks with fetal death before or during delivery; thereafter, the definition was expanded to include deliveries ≥22 + 0 gestational weeks. Neonatal death was defined as a liveborn who died 0 to 27 days after birth. Major birth defects at birth were defined according to the European Concerted Action on Congenital Anomalies and Twins classification (EUROCAT) (29).

### Covariates

Covariates included year of treatment, the woman’s age, parity, previous IVF children, country of birth, maternal smoking, cause of infertility, educational level, number of previously failed fresh and frozen embryo transfer cycles, number of retrieved oocytes, fertilization method and type of embryo transfer, and number of embryos transferred.

### Statistical analyses

Descriptive statistics are given by number and percentage and presented for the WHO’s six BMI classes. Crude risk ratios (RRs) and adjusted RR with 95% confidence intervals (CIs) were obtained using general estimation equation models, adjusting for dependence within each woman. We estimated RRs with 95% CIs for outcomes in subpopulations 1 and 2 in underweight women, in overweight women, in women with obesity class I, and in women with obesity classes II and III combined (due to small numbers in class III), using women with normal weight as reference.

For subpopulation 1, adjustments were made for year of treatment (continuous), the woman’s age (years, continuous), the woman’s country of birth (Sweden/other European/outside Europe), the woman’s educational level (≤9, 10–12, >13 years), fertilization method (IVF/ICSI), number of previous failed started fresh IVF cycles (continuous), and number of previous IVF children (continuous). The multifetal pregnancy RR was, in addition, adjusted for the number of embryos transferred (continuous). We did not adjust for culture duration, resulting in cleavage stage transfer or blastocyst transfer or fresh or frozen embryo transfer since these variables may be associated with the exposure (BMI). Neither did we adjust (except for multiple birth rates) for the number of embryos transferred. In Sweden, single embryo transfer is used in a majority of all cycles and double embryo transfer is used only when only poor quality embryos are available, which also may be associated with the exposure (BMI).

For subpopulation 2, adjustments were made for the year of treatment (continuous), maternal age (continuous), parity (0/≥1), maternal country of birth (Sweden/other European/outside Europe), maternal educational level (≤9, 10–12, >13 years), maternal smoking (yes/no), fertilization method (IVF/ICSI), and type of embryo transfer (fresh/frozen).

Records with missing information on BMI were excluded since BMI was the variable of interest.

In subpopulation 1, a sensitivity analysis was performed excluding treatments carried out between 2007 and 2009 where BMI was often missing. Furthermore, characteristics and outcome by availability of BMI information are displayed in the Supplementary Material for subpopulation 1.

A *p*-value less than 0.05 was considered significant. Data were analyzed with the statistical package SPSS version 27 (IBM Corp., Armonk, NY, USA).

## Results

### Live birth

The association between different BMI classes and live birth rate and cumulative live birth rate was analyzed in 126,620 started fresh IVF/ICSI cycles and subsequent frozen embryo transfers. In this subpopulation, 2.4% of the cycles included underweight women, 62% normal weight, 26% overweight, 8.7% obesity class I, 1.1% obesity class II, and 0.05% obesity class III. The descriptive characteristics of subpopulation 1 are shown in **Table 1**. Higher female age was related to higher BMI. Data on the number of started fresh cycles per year by women’s BMI class are shown in **Supplementary Table S1**.

**Table 1:** Characteristics of women by started fresh IVF cycle and BMI class.

		BMI class												Total	
		<18.5		18.5–24.9		25–29.9		30–34.9		35–39.9		≥40			
		*N* = 3,084		*N* = 78,524		*N* = 32,513		*N* = 11,036		*N* = 1,397		*N* = 66		*N* = 126,620	
		*n*	(%)	*n*	(%)	*n*	(%)	*n*	(%)	*n*	(%)	*N*	(%)	*n*	(%)
Woman’s age (years)															
	<30	703	(22.8)	13,744	(17.5)	6,105	(18.8)	2,166	(19.6)	223	(16.0)	13	(19.7)	22,954	(18.1)
	30–34	1,151	(37.3)	27,592	(35.1)	10,169	(31.3)	3,249	(29.4)	390	(27.9)	11	(16.7)	42,562	(33.6)
	35–37	648	(21.0)	17,863	(22.7)	7,376	(22.7)	2,391	(21.7)	291	(20.8)	9	(13.6)	28,578	(22.6)
	38–39	353	(11.4)	10,745	(13.7)	4,994	(15.4)	1,880	(17.0)	253	(18.1)	11	(16.7)	18,236	(14.4)
	≥40	229	(7.4)	8,580	(10.9)	3,869	(11.9)	1,350	(12.2)	240	(17.2)	22	(33.3)	14,290	(11.3)
Parity (previous children)															
	No	2,385	(77.3)	60,519	(77.1)	25,120	(77.3)	8,557	(77.5)	1,080	(77.3)	54	(81.8)	97,715	(77.2)
	Yes	699	(22.7)	18,005	(22.9)	7,393	(22.7)	2,479	(22.5)	317	(22.7)	12	(18.2)	28,905	(22.8)
Country of birth															
	Sweden	2,149	(69.7)	61,265	(78.0)	24,233	(74.5)	8,264	(74.9)	1,051	(75.2)	55	(83.3)	97,017	(76.6)
	Other European	359	(11.6)	7,383	(9.4)	2,875	(8.8)	856	(7.8)	123	(8.8)	0	(0.0)	11,596	(9.2)
	Outside Europe	576	(18.7)	9,876	(12.6)	5,405	(16.6)	1,916	(17.4)	223	(16.0)	11	(16.7)	18,007	(14.2)
Cause of infertility															
	Male factor	740	(24.0)	20,193	(25.7)	8,852	(27.2)	2,706	(24.5)	329	(23.6)	26	(39.4)	32,846	(25.9)
	Tubal factor	183	(5.9)	4,640	(5.9)	2,426	(7.5)	806	(7.3)	84	(6.0)	2	(3.0)	8,141	(6.4)
	Endometriosis	189	(6.1)	4,677	(6.0)	1,881	(5.8)	563	(5.1)	42	(3.0)	3	(4.5)	7,355	(5.8)
	Female factor, not specified	680	(22.0)	18,658	(23.8)	7,022	(21.6)	2,217	(20.1)	257	(18.4)	4	(6.1)	28,838	(22.8)
	PCOS	133	(4.3)	3,121	(4.0)	2,168	(6.7)	1,211	(11.0)	159	(11.4)	8	(12.1)	6,800	(5.4)
	Anovulation	359	(11.6)	7,382	(9.4)	3,695	(11.4)	2,051	(18.6)	268	(19.2)	9	(13.6)	13,764	(10.9)
	Unexplained or not known	1,066	(34.6)	26,693	(34.0)	10,216	(31.4)	3,288	(29.8)	475	(34.0)	22	(33.3)	41,760	(33.0)
Educational level (years)															
	≤9	221	(7.2)	4,940	(6.3)	3,301	(10.2)	1,457	(13.2)	200	(14.3)	13	(19.7)	10,132	(8.0)
	10–12	720	(23.3)	18,281	(23.3)	9,724	(29.9)	4,002	(36.3)	543	(38.9)	25	(37.9)	33,295	(26.3)
	≥13	2,101	(68.1)	54,809	(69.8)	19,243	(59.2)	5,504	(49.9)	642	(46.0)	28	(42.4)	82,327	(65.0)
	Not known^a^	42	(1.4)	494	(0.6)	245	(0.8)	73	(0.7)	12	(0.9)	0	(0.0)	866	(0.7)
Previous failed started fresh cycles															
	0	1,729	(56.1)	42,083	(53.6)	17,043	(52.4)	5,867	(53.2)	767	(54.9)	45	(68.2)	67,534	(53.3)
	1	704	(22.8)	18,803	(23.9)	8,057	(24.8)	2,846	(25.8)	355	(25.4)	13	(19.7)	30,778	(24.3)
	2	352	(11.4)	9,272	(11.8)	4,014	(12.3)	1,360	(12.3)	172	(12.3)	6	(9.1)	15,176	(12.0)
	≥3	299	(9.7)	8,366	(10.7)	3,399	(10.5)	963	(8.7)	103	(7.4)	2	(3.0)	13,132	(10.4)
Previous children after IVF															
	No	2,687	(87.1)	69,238	(88.2)	29,123	(89.6)	9,932	(90.0)	1,286	(92.1)	65	(98.5)	112,331	(88.7)
	Yes	397	(12.9)	9,286	(11.8)	3,390	(10.4)	1,104	(10.0)	111	(7.9)	1	(1.5)	14,289	(11.3)
Years when started fresh IVF cycle															
	2007–2011	1,061	(34.4)	27,542	(35.1)	10,412	(32.0)	3,300	(29.9)	545	(39.0)	41	(62.1)	42,901	(33.9)
	2012–2015	1,008	(32.7)	25,750	(32.8)	10,522	(32.4)	3,642	(33.0)	455	(32.6)	18	(27.3)	41,395	(32.7)
	2016–2019	1,015	(32.9)	25,232	(32.1)	11,579	(35.6)	4,094	(37.1)	397	(28.4)	7	(10.6)	42,324	(33.4)
Fertilization method^b^															
	IVF	1,191	(40.8)	32,502	(43.4)	12,244	(39.8)	4,031	(39.1)	547	(41.9)	15	(23.1)	50,530	(42.0)
	ICSI	1,123	(38.5)	29,028	(38.8)	12,861	(41.8)	4,169	(40.4)	492	(37.7)	31	(47.7)	47,704	(39.7)
Retrieved oocytes															
	No oocytes retrieved	168	(5.4)	3,714	(4.7)	1,740	(5.4)	726	(6.6)	93	(6.7)	1	(1.5)	6,442	(5.1)
	<5	480	(15.6)	12,598	(16.0)	5,769	(17.7)	2,334	(21.1)	293	(21.0)	20	(30.3)	21,494	(17.0)
	5–9	1,130	(36.6)	28,325	(36.1)	12,016	(37.0)	4,026	(36.5)	521	(37.3)	27	(40.9)	46,045	(36.4)
	10–19	1,151	(37.3)	29,412	(37.5)	11,481	(35.3)	3,464	(31.4)	429	(30.7)	17	(25.8)	45,954	(36.3)
	≥20	155	(5.0)	4,475	(5.7)	1,507	(4.6)	486	(4.4)	61	(4.4)	1	(1.5)	6,685	(5.3)
Presence and type of fresh ET															
	No fresh ET	690	(22.4)	14,997	(19.1)	6,695	(20.6)	2,561	(23.2)	324	(23.2)	20	(30.3)	25,287	(20.0)
	Fresh SET	1,979	(64.2)	49,843	(63.5)	20,177	(62.1)	6,682	(60.5)	839	(60.1)	37	(56.1)	79,557	(62.8)
	Fresh DET	415	(13.5)	13,684	(17.4)	5,641	(17.3)	1,793	(16.2)	234	(16.8)	9	(13.6)	21,776	(17.2)
Number of frozen ETs per OA															
	No frozen ET	2,322	(75.3)	58,990	(75.1)	24,920	(76.6)	8,506	(77.1)	1,126	(80.6)	54	(81.8)	95,918	(75.8)
	1–2 frozen ETs	682	(22.1)	17,543	(22.3)	6,856	(21.1)	2,297	(20.8)	238	(17.0)	11	(16.7)	27,627	(21.8)
	3–4 frozen ETs	73	(2.4)	1,853	(2.4)	694	(2.1)	219	(2.0)	33	(2.4)	1	(1.5)	2,873	(2.3)
	5 frozen ET	7	(0.2)	138	(0.2)	43	(0.1)	14	(0.1)	0	(0.0)	0	(0.0)	202	(0.2)

Started fresh IVF cycle: a fresh IVF cycle where at least one dose of gonadotrophins was administered.

DET, double embryo transfer; ET, embryo transfer; ICSI, intracytoplasmic sperm injection; IVF, *in-vitro* fertilization; OA, oocyte aspiration; PCOS, polycystic ovary syndrome; SET, single embryo transfer.

a In the statistical analysis, missing values were replaced by the overall mean.

b Fewer numbers compared with the started cycles since the fertilization method (IVF/ICSI) is only known for cycles resulting in ET.

**Figure 1:**
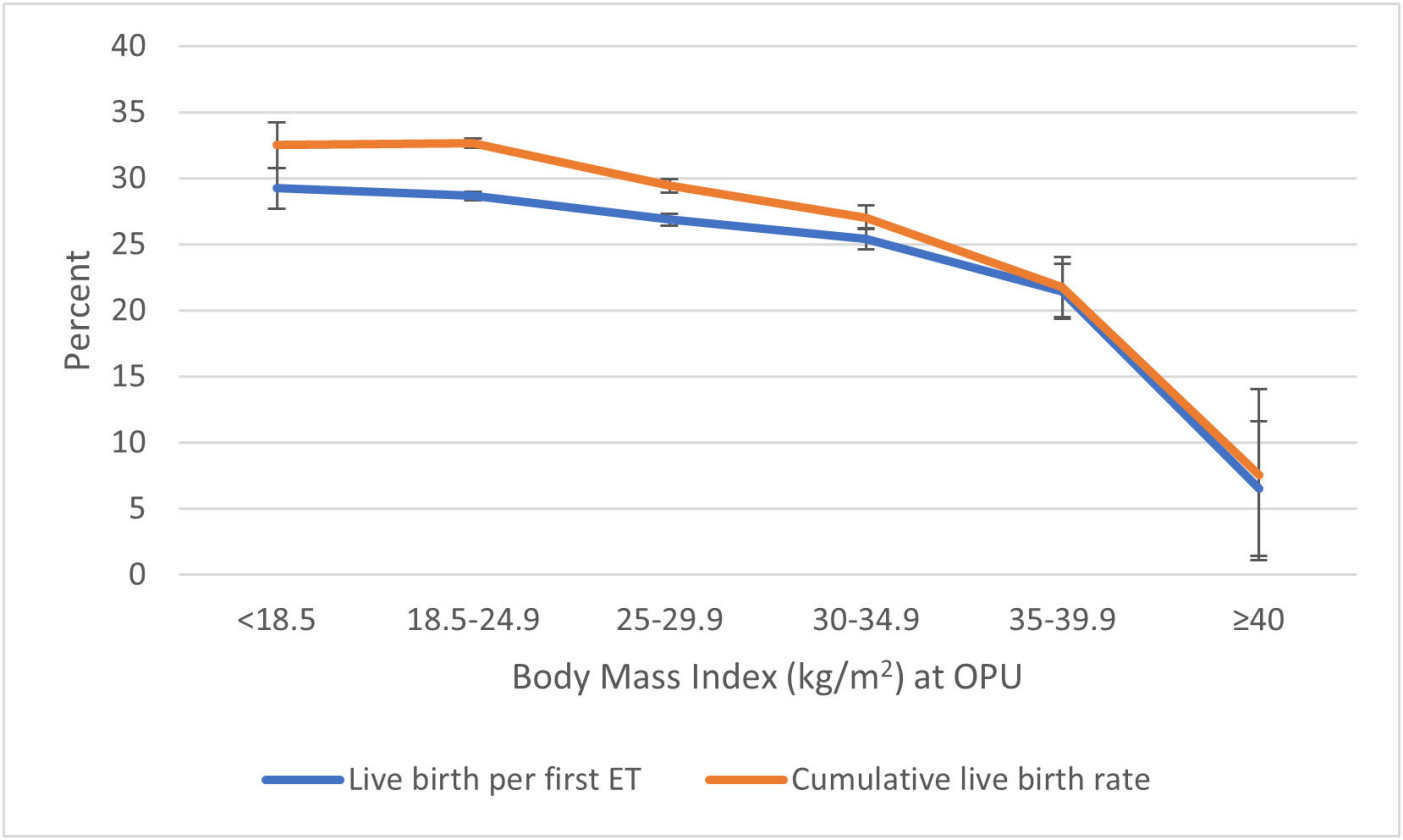
Live birth per first embryo transfer and cumulative live birth^†^ per started fresh IVF cycle in relation to BMI class. ^†^Cumulative live birth rate: the number of deliveries with at least one live born child (singleton or multiple) per started fresh IVF cycle, including one fresh and/or all frozen embryo transfers within 1 year, until one delivery with a live birth or until all embryos were used, whichever occurred first. BMI, body mass index.

The cumulative live birth rate decreased with increasing BMI (**Tables 2**, **3**; **Figure 1**). The cumulative live birth rate was 29.4% in overweight women and 32.6% in normal-weight women (adjusted RR 0.92; 95% CI 0.90–0.93). The cumulative live birth rate for women in obesity class I was 27.0% (adjusted RR 0.86; 95% CI 0.83–0.88) and for women in obesity class II 21.8% and obesity class III 7.6% (adjusted RR 0.70; 95% CI 0.63–0.77, class II + III). The adjustment only marginally changed the RRs.

**Table 2:** Live birth per started fresh IVF cycle, cumulative live birth per started fresh cycle, live birth per fresh embryo transfer, and live birth per first embryo transfer in relation to BMI class.

BMI (kg/m²)	LB per started fresh cycle^a^		CLB per started fresh cycle^b^		Started fresh cycles	LB per fresh ET		Fresh ET	LB per first ET		First ET
	*n*	(%)	*n*	(%)	*n*	*n*	(%)	*n*	*n*	(%)	*n*
<18.5	697	(22.6)	1,003	(32.5)	3,084	697	(29.1)	2,394	748	(29.3)	2,557
18.5–24.9	17,914	(22.8)	25,636	(32.6)	78,524	17,906	(28.2)	63,527	19,174	(28.7)	66,875
25–29.9	6,838	(21.0)	9,567	(29.4)	32,513	6,837	(26.5)	25,818	7,307	(26.9)	27,187
30–34.9	2,126	(19.3)	2,985	(27.0)	11,036	2,124	(25.1)	8,475	2,293	(25.4)	9,019
35–39.9	231	(16.5)	304	(21.8)	1,397	231	(21.5)	1,073	243	(21.4)	1,134
≥40	3	(4.5)	5	(7.6)	66	3	(6.5)	46	3	(6.5)	46

a Started fresh IVF cycle: a fresh IVF cycle where at least one dose of gonadotrophins was administered.

b Cumulative live birth rate: the number of deliveries with at least one live born child (singleton or multiple) per started fresh IVF cycle, including one fresh and/or all frozen embryo transfers within 1 year, until one delivery with a live birth or until all embryos were used, whichever occurred first.

BMI, body mass index; LB, live birth; CLB, cumulative live birth; ET, embryo transfer.

**Table 3:** Cumulative live birth per started fresh IVF cycle in relation to BMI class.

BMI (kg/m²)	CLBR		Started fresh IVF cycles	Risk ratio		Adjusted risk ratio^a^		*p*-value
	*n*	(%)	*N*	RR	95% CI	ARR	95% CI	
<18.5	1,003	(32.5)	3,084	1.0	0.94–1.05	0.96	0.91–1.01	0.108
18.5–24.9	25,636	(32.6)	78,524	1.0	Reference	1.0	reference	
25–29.9	9,567	(29.4)	32,513	0.90	0.88–0.91	0.92	0.90–0.93	<0.000
30–34.9	2,985	(27.0)	11,036	0.82	0.80–0.85	0.86	0.83–0.88	<0.000
≥35	309	(21.1)	1,463	0.64	0.58–0.71	0.70	0.63–0.77	<0.000

Cumulative live birth rate: the number of deliveries with at least one live born child (singleton or multiple) per started fresh IVF cycle, including one fresh and/or all frozen embryo transfers within 1 year, until one delivery with a live birth or until all embryos were used, whichever occurred first. Started fresh IVF cycle: a fresh IVF cycle where at least one dose of gonadotrophins was administered.

ARR, adjusted risk ratio; BMI, body mass index; CI, confidence interval; CLBR, cumulative live birth rate; n, number; RR, risk ratio.

a Adjusted for the year of treatment (continuous), maternal age (continuous), country of birth (Sweden/other European/outside Europe), educational level (ordinal), type of IVF treatment (IVF/ICSI), number of previous failed fresh cycles (continuous), and number of previous IVF children (continuous).

Live birth per started fresh cycle was 22.6% in underweight women, 22.8% in normal-weight women, 21.0% in overweight women, 19.3% in women in obesity class I, 16.5% in women in obesity class II, and 4.5% in women with obesity class III. The live birth per first and per fresh embryo transfer decreased correspondingly with increasing BMI (**Table 2**; **Figure 1**).

#### Sensitivity analysis and dropout analysis

After the exclusion of the years 2007–2009, the association between BMI and cumulative live birth rate changed only marginally.

Dropout analyses by the availability of BMI information for subpopulation 1 are presented in **Supplementary Tables S2**, **S3**. Cumulative live birth rates were slightly lower in women with missing BMI.

#### Miscarriage

The risk of miscarriage increased significantly with increasing BMI (**Supplementary Table S4**). In overweight women, the rate of miscarriage was 18.9% compared with 17.1% in normal-weight women (adjusted RR 1.10; 95% CI 1.05–1.15). In women in obesity class I and class II + III, the rate of miscarriage was 21.1% and 25.9% (adjusted RR 1.21; 95% CI 1.13–1.29 and adjusted RR 1.36; 95% CI 1.16–1.60).

#### Multifetal pregnancies

The risk of multifetal pregnancy per delivery after first embryo transfer increased in women in obesity class I compared with normal-weight women: 5.4% vs. 4.3% (adjusted RR 1.33; 95% CI 1.12–1.58) (**Supplementary Table S5**).

### Maternal and perinatal outcomes

The association between BMI class and maternal and perinatal outcomes was analyzed in 58,187 singleton deliveries. In this population, 2.2% of the women were underweight, 64.7% normal weight, 24.4% overweight, 7.5% obesity class I, 1.0% obesity class II, and 0.1% obesity class III. The descriptive characteristics of subpopulation 2 are shown in **Table 4**. Data are shown in **Supplementary Table S6** on the number of IVF treatments leading to delivery per year in relation to the BMI of women.

**Table 4:** Maternal and treatment characteristics by maternal BMI class for women with singleton deliveries after IVF treatment, 2002–2020.

		BMI class													Total	
		<18.5		18.5–24.9		25–29.9		30–34.9		35–39.9		≥40				
		*n* = 1,289		*n* = 37,701		*n* = 14,211		*n* = 4,352		*n* = 575		*n* = 59			58,187	
		*n*	(%)	*n*	(%)	*n*	(%)	*n*	(%)	*n*	(%)	*n*	(%)	*n*		(%)
Woman’s age (years)																
	<35	964	(74.8)	26,588	(70.5)	9,645	(67.9)	2,914	(67.0)	385	(67.0)	40	(67.8)	40,536		(69.7)
	≥35	325	(25.2)	11,113	(29.5)	4,566	(32.1)	1,438	(33.0)	190	(33.0)	19	(32.2)	17,651		(30.3)
Parity (previous children)																
	No	883	(68.5)	25,169	(66.8)	9,368	(65.9)	2,889	(66.4)	383	(66.6)	39	(66.1)	38,731		(66.6)
	Yes	406	(31.5)	12,532	(33.2)	4,843	(34.1)	1,463	(33.6)	192	(33.4)	20	(33.9)	19,456		(33.4)
Country of birth																
	Sweden	935	(72.5)	30,861	(81.9)	11,298	(79.5)	3,481	(80.0)	486	(84.5)	46	(78.0)	47,107		(81.0)
	Other European	155	(12.0)	3,290	(8.7)	1,142	(8.0)	311	(7.1)	44	(7.7)	6	(10.2)	4,948		(8.5)
	Outside Europe	199	(15.4)	3,548	(9.4)	1,768	(12.4)	560	(12.9)	45	(7.8)	7	(11.9)	6,127		(10.5)
	Not known^a^	0	(0.0)	2	(0.0)	3	(0.0)	0	(0.0)	0	(0.0)	0	(0.0)	5		(0.0)
Maternal smoking																
	No	1,211	(93.9)	35,651	(94.6)	13,392	(94.2)	4,036	(92.7)	523	(91.0)	50	(84.7)	54,863		(94.3)
	Yes	20	(1.6)	490	(1.3)	315	(2.2)	127	(2.9)	23	(4.0)	8	(13.6)	983		(1.7)
	Not known^a^	58	(4.5)	1,560	(4.1)	504	(3.5)	189	(4.3)	29	(5.0)	1	(1.7)	2,341		(4.0)
Cause of infertility																
	Male factor	304	(23.6)	10,291	(27.3)	3,779	(26.6)	1,076	(24.7)	163	(28.3)	19	(32.2)	15,632		(26.9)
	Tubal factor	74	(5.7)	1,558	(4.1)	668	(4.7)	221	(5.1)	30	(5.2)	1	(1.7)	2,552		(4.4)
	Endometriosis	67	(5.2)	1,563	(4.1)	503	(3.5)	153	(3.5)	15	(2.6)	0	(0.0)	2,301		(4.0)
	Female factor not specified	235	(18.2)	6,700	(17.8)	2,161	(15.2)	622	(14.3)	59	(10.3)	3	(5.1)	9,780		(16.8)
	PCOS	43	(3.3)	1,310	(3.5)	715	(5.0)	331	(7.6)	42	(7.3)	3	(5.1)	2,444		(4.2)
	Anovulation	118	(9.2)	2,936	(7.8)	1,260	(8.9)	571	(13.1)	86	(15.0)	8	(13.6)	4,979		(8.6)
	Unexplained or not known	527	(40.9)	15,562	(41.3)	6,119	(43.1)	1,789	(41.1)	239	(41.6)	31	(52.5)	24,267		(41.7)
Pre-pregnancy morbidity																
	Chronic hypertension	1	(0.1)	118	(0.3)	130	(0.9)	87	(2.0)	14	(2.4)	1	(1.7)	351		(0.6)
	Diabetes, type 1 or 2	4	(0.3)	195	(0.5)	143	(1.0)	72	(1.7)	10	(1.7)	2	(3.4)	426		(0.7)
Educational level (years)																
	≤9	93	(7.2)	1,970	(5.2)	1,283	(9.0)	473	(10.9)	59	(10.3)	9	(15.3)	3,887		(6.7)
	10–12	293	(22.7)	9,080	(24.1)	4,441	(31.3)	1,684	(38.7)	276	(48.0)	34	(57.6)	15,808		(27.2)
	≥13	891	(69.1)	26,484	(70.2)	8,408	(59.2)	2,171	(49.9)	236	(41.0)	16	(27.1)	38,206		(65.7)
	Not known	12	(0.9)	167	(0.4)	79	(0.6)	24	(0.6)	4	(0.7)	0	(0.0)	286		(0.5)
Years when started fresh IVF cycle^b^																
	2002–2010	378	(29.3)	13,758	(36.5)	5,327	(37.5)	1,629	(37.4)	326	(56.7)	49	(83.1)	21,467		(36.9)
	2011–2020	911	(70.7)	23,943	(63.5)	8,884	(62.5)	2,723	(62.6)	249	(43.3)	10	(16.9)	36,720		(63.1)
Fertilization method																
	IVF	798	(61.9)	22,878	(60.7)	8,904	(62.7)	2,724	(62.6)	351	(61.0)	33	(55.9)	35,688		(61.3)
	ICSI	491	(38.1)	14,823	(39.3)	5,307	(37.3)	1,628	(37.4)	224	(39.0)	26	(44.1)	22,499		(38.7)
Treatment type																
	Fresh	819	(63.5)	24,163	(64.1)	9,546	(67.2)	2,934	(67.4)	419	(72.9)	37	(62.7)	37,918		(65.2)
	Frozen	470	(36.5)	13,538	(35.9)	4,665	(32.8)	1,418	(32.6)	156	(27.1)	22	(37.3)	20,269		(34.8)
Number of culture days																
	1–3	779	(60.4)	24,198	(64.2)	9,361	(65.9)	2,923	(67.2)	437	(76.0)	52	(88.1)	37,750		(64.9)
	4–8	510	(39.6)	13,503	(35.8)	4,850	(34.1)	1,429	(32.8)	138	(24.0)	7	(11.9)	20,437		(35.1)
Number of embryos transferred																
	1	1,143	(88.7)	32,195	(85.4)	11,912	(83.8)	3,648	(83.8)	452	(78.6)	51	(86.4)	49,401		(84.9)
	2	146	(11.3)	5,502	(14.6)	2,296	(16.2)	704	(16.2)	123	(21.4)	8	(13.6)	8,779		(15.1)
	3	0	(0.0)	4	(0.0)	3	(0.0)	0	(0.0)	0	(0.0)	0	(0.0)	7		(0.0)

a In the statistical analysis, missing values were replaced by the overall mean.

b Started fresh IVF cycle: a fresh IVF cycle where at least one dose of gonadotrophins was administered.

ICSI, intracytoplasmic sperm injection; IVF, *in-vitro* fertilization; PCOS, polycystic ovary syndrome.

#### Maternal outcomes

**Figure 2:**
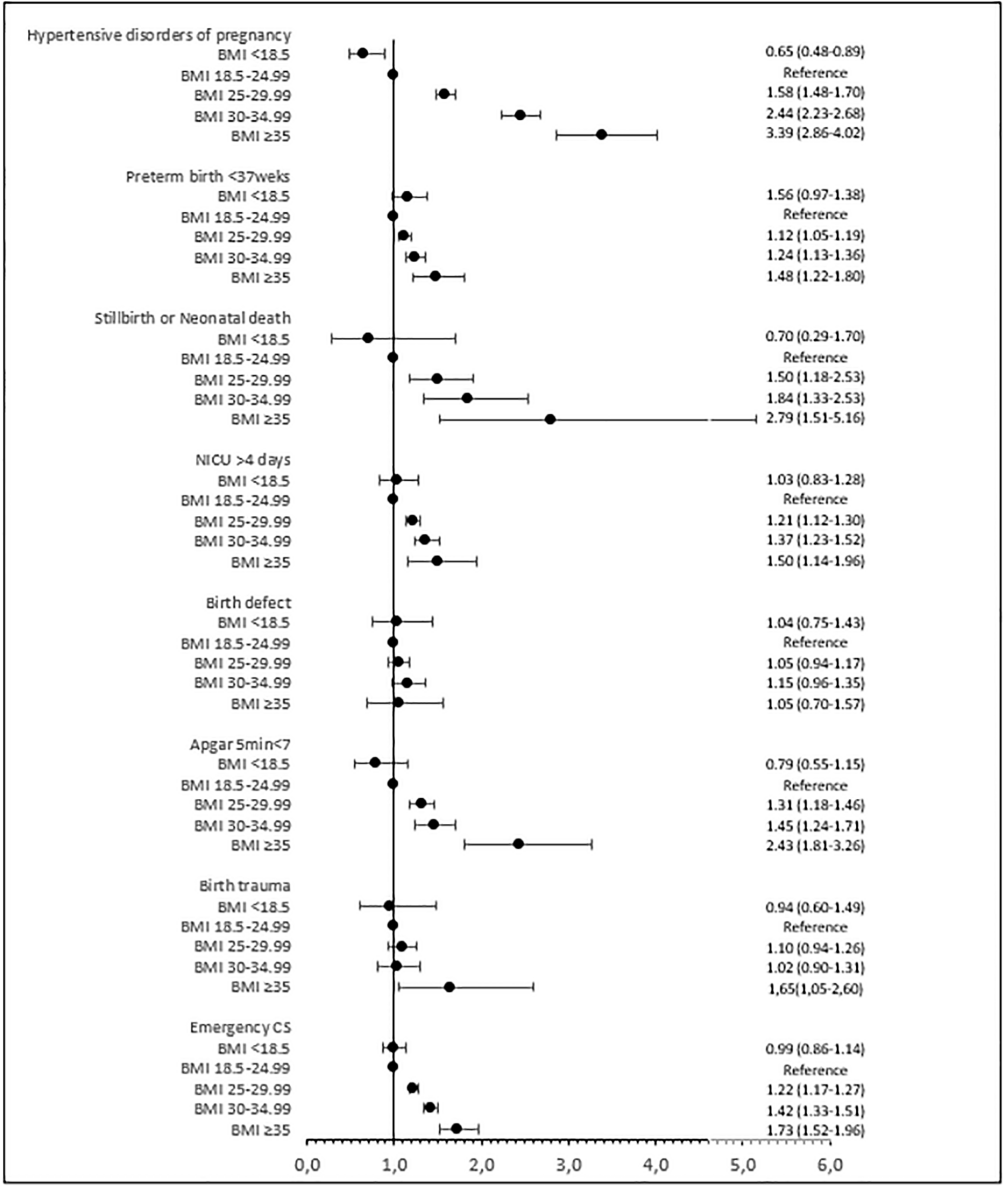
Adjusted risk ratios (95% confidence intervals) for maternal and perinatal outcomes by BMI class. Adjusted for year of treatment (continuous), maternal age (continuous), maternal country of birth (Sweden/other European/outside Europe), maternal educational level (ordinal), fertilization method (IVF/ICSI), type of embryo transfer (fresh/frozen), parity (continuous), and maternal smoking (yes/no). BMI, body mass index; CS, cesarean section; NICU, neonatal intensive care unit.

Maternal outcomes are shown in **Table 5** and **Figure 2**. The risk of hypertensive disorders during pregnancy increased significantly with increasing BMI. In overweight women, the risk of hypertensive disorders of pregnancy was 7.8% compared with 4.6% in normal-weight women (adjusted RR 1.58; 95% CI 1.48–1.70). For women in obesity classes I, II, and III, the risk of hypertensive disorders of pregnancy was 12.5%, 17.9%, and 20.3% (adjusted RR 2.44; 95% CI 2.23–2.68 for class I and adjusted RR 3.39; 95% CI 2.86–4.02 for class II + III). The risk of gestational diabetes and emergency cesarean section followed the same pattern.

**Table 5:** Maternal and perinatal outcomes by BMI class.

			BMI class												Total		
			<18.5		18.5–24.9		25–29.9		30–34.9		35–39.9		≥40				
			*n* = 1,289		*n* = 37,701		*n* = 14,211		*n* = 4,352		*n* = 575		*n* = 59		58,187		
			*n*	(%)	*n*	(%)	*N*	(%)	*n*	(%)	*n*	(%)	*n*	(%)	*n*	(%)	
Pregnancy complications																	
	HDP		38	(2.9)	1,740	(4.6)	1,102	(7.8)	542	(12.5)	103	(17.9)	12	(20.3)	3,537		(6.1)
	Gestational diabetes		8	(0.6)	413	(1.1)	388	(2.7)	244	(5.6)	52	(9.0)	8	(13.6)	1,113		(1.9)
Mode of delivery																	
	Vaginal		878	(68.1)	25,058	(66.5)	8,906	(62.7)	2,572	(59.1)	332	(57.7)	28	(47.5)	37,774		(64.9)
	Elective CS		135	(10.5)	3,908	(10.4)	1,569	(11.0)	544	(12.5)	61	(10.6)	5	(8.5)	6,222		(10.7)
	Emergency CS		159	(12.3)	5,044	(13.4)	2,453	(17.3)	900	(20.7)	152	(26.4)	20	(33.9)	8,728		(15.0)
	VE/forceps		117	(9.1)	3,691	(9.8)	1,283	(9.0)	336	(7.7)	30	(5.2)	6	(10.2)	5,463		(9.4)
Delivery complications																	
	Postpartum hemorrhage		95	(7.4)	2,872	(7.6)	1,047	(7.4)	344	(7.9)	46	(8.0)	5	(8.5)	4,409		(7.6)
	Shoulder dystocia		<3^a^	(<0.2)	73	(0.2)	36	(0.3)	15	(0.3)	0	(0.0)	0	(0.0)	126		(0.2)
Perinatal outcomes																	
	Males		663	(51.4)	19,362	(51.4)	7,378	(51.9)	2,251	(51.7)	303	(52.7)	34	(57.6)	29,991		(51.5)
	Females		626	(48.6)	18,339	(48.6)	6,833	(48.1)	2,101	(48.3)	272	(47.3)	25	(42.4)	28,196		(48.5)
	Birth weight <1,500 g		16	(1.2)	370	(1.0)	209	(1.5)	90	(2.1)	8	(1.4)	<3^a^	(<5.1)	695		(1.2)
	Birth weight <2,500 g		86	(6.7)	1,763	(4.7)	738	(5.2)	277	(6.4)	29	(5.0)	7	(11.9)	2,900		(5.0)
	Birth weight >4,500 g		12	(0.9)	940	(2.5)	607	(4.3)	218	(5.0)	49	(8.5)	4	(6.8)	1,830		(3.1)
	Gestational age <32 weeks		13	(1.0)	400	(1.1)	218	(1.5)	88	(2.0)	9	(1.6)	3	(5.1)	731		(1.3)
	Gestational age <37 weeks		100	(7.8)	2,388	(6.3)	1,071	(7.5)	388	(8.9)	57	(9.9)	9	(15.3)	4,013		(6.9)
	SGA		84	(6.5)	1,597	(4.2)	592	(4.2)	195	(4.5)	26	(4.5)	6	(10.2)	2,500		(4.3)
	LGA		20	(1.6)	1,285	(3.4)	923	(6.5)	364	(8.4)	76	(13.2)	6	(10.2)	2,674		(4.6)
	Stillborn		<3^a^	(<0.2)	91	(0.2)	49	(0.3)	27	(0.6)	5	(0.9)	<3^a^	(<5.1)	174		(0.3)
	Neonatal death, days 0–27		<3^a^	(<0.2)	44	(0.1)	31	(0.2)	8	(0.2)	<3^a^	(<0.5)	0	(0.0)	86		(0.1)
	Apgar <4, 5 min		12	(0.9)	345	(0.9)	189	(1.3)	66	(1.5)	11	(1.9)	<3^a^	(<5.1)	625		(1.1)
	Apgar <7, 5 min		25	(1.9)	793	(2.1)	425	(3.0)	146	(3.4)	32	(5.6)	5	(8.5)	1,426		(2.5)
	Major birth defects		36	(2.8)	924	(2.5)	390	(2.7)	139	(3.2)	19	(3.3)	3	(5.1)	1,511		(2.6)
	NICU >4 days		68	(5.3)	1,827	(4.8)	934	(6.6)	340	(7.8)	37	(6.4)	6	(10.2)	3,212		(5.5)
	Birth trauma		19	(1.5)	579	(1.5)	241	(1.7)	71	(1.6)	16	(2.8)	3	(5.1)	929		(1.6)

a The exact numbers are not reported to ensure the anonymity of the participants.

BMI, body mass index; CS, cesarean section; HDP, hypertensive disorders of pregnancy; LGA, large for gestational age; n, number; NICU, neonatal intensive care unit; SGA, small for gestational age; VE, vacuum extraction.

#### Perinatal outcomes

The risk of preterm birth at less than 37 gestational weeks increased significantly with increasing BMI (**Table 5**; **Figure 2**). In overweight women, the risk of preterm birth at less than 37 gestational weeks was 7.5%, and in normal-weight women, this risk was 6.3% (adjusted RR 1.12; 95% CI 1.05–1.19). In women in obesity classes I, II, and III, the risk of preterm birth at less than 37 gestational weeks was 8.9%, 9.9%, and 15.3% (adjusted RR 1.24; 95% CI 1.13–1.36 for class I and adjusted RR 1.48; 95% CI 1.22–1.80 for class II + III). The risk of stillbirth or neonatal death, low Apgar score, and admission to a neonatal intensive care unit was significantly increased in overweight women and those in obesity class I and class II + III compared with normal-weight women, and the risk increased with BMI class. The risk of macrosomia and large for gestational age followed the same pattern. The risk of any birth trauma was increased in women in obesity class II + III.

## Discussion

This large, national population-based cohort study found that the live birth rate after IVF, assessed as live birth per started fresh cycle, per first embryo transfer, and as cumulative live birth rate, including fresh and frozen cycles from a single started fresh cycle, decreased with maternal overweight and obesity in a dose-dependent manner. The cumulative live birth rate decreased from 32.6% in normal-weight women to 29.4% in overweight women and to 27.0%, 21.8%, and 7.6% in women in obesity classes I, II, and III.

Furthermore, adverse maternal and perinatal outcomes increased progressively with increasing BMI. The rate of hypertensive disorders of pregnancy increased from 4.6% in normal-weight women to 7.6% in overweight women and to 12.5%, 17.9%, and 20.3% in women in obesity classes I, II, and III, and the rate for preterm birth increased correspondingly, from 6.3% in normal-weight women to 7.5%, 8.9%, 9.9%, and 15.3% in overweight women and women in obesity classes I, II, and III.

The strengths of this study were the collection of data in an unselected and complete national population, analysis of a large number of IVF cycles over many years, and inclusion of delivery outcomes during an extended period. Due to the unique Swedish personal identification number, data from several high-quality registers could be cross-linked. The linkage enabled the assessment of the association between BMI and outcomes after IVF regarding both the number of live births and the maternal and perinatal consequences after adjustment for relevant confounders.

The main limitation of this study is that BMI data were missing in 16% of fresh IVF cycles in the analysis of live birth rates. The percentage of cycles with missing BMI was higher during the first years; however, a sensitivity analysis excluding these first years showed cumulative live birth rates similar to those in the main analysis. We chose to set a time limit, 1 year after starting fresh cycles, during which women’s frozen cycles could contribute to cumulative live birth rate, regardless of if frozen embryos were still available. This was done to give each woman the same time to reach a live birth, which otherwise would have differed markedly between women. To use the original definition of cumulative live birth, that all embryos from a single cycle should be used, would almost be impossible, since the Swedish law allows embryos to be frozen for 10 years. Furthermore, most embryos left in the freezer at the end of this period were from couples that already had achieved a live birth, thus not affecting the cumulative live birth rate to any major extent.

The number of cycles in women with a BMI above 40 kg/m² was limited in this study due to a low prevalence of extreme obesity in Sweden and the BMI limit of less than 35 kg/m^2^ to access IVF treatment in publicly funded clinics. Hence, the results in the highest BMI group should be taken with caution.

A strength in this study is the reporting of cumulative live birth rate and live birth after the first embryo transfer (fresh or frozen) reflecting the current embryo transfer policy with increased rates of frozen embryo transfers also as first transfer. A large study from the United States analyzed live birth rate only after fresh embryo transfer and a few perinatal outcomes and found a decreased live birth rate and higher rates of preterm birth and low birth weight in obese women (18). Other large studies that have analyzed live birth rates in relation to BMI, either per fresh cycle or cumulatively, have found decreased live birth rates in obese women, up to ≥50 kg/m², compared with normal-weight women (30, 31). The cumulative live birth rate in Sweden during this time period was lower compared with, e.g., United States data, presented by Goldman et al. (30). However, there were several differences between the study of Goldman and the present study. Goldman included only first-time IVF cycles, while the present study included all cycles performed in Sweden during the actual time period, e.g., 10% were cycles in women with three or more failed cycles. The number of failed cycles is a negative predictor for live birth after IVF. Goldman et al. covered the years 2014–2015, while the present study included the time period 2007–2020. Thus, our study included an earlier time period with less good results. A large Swedish registry study, including all cycles 2007–2017, showed that the cumulative live birth rate has increased over time, accompanied by a higher rate of blastocyst transfers performed every year. The cumulative live birth rate was 27.0% in 2007 and increased to 36.3% in 2017 when assessed per oocyte retrieval (32). In 2019, this figure was 43.2% (20).

The multiple birth rate in the study by Goldman et al. was high, between 10% and 34%, indicating that several embryos have been transferred per cycle, while the Swedish data included mainly single embryo transfers, giving a low multiple birth rate, between 4.5% and 6.3%. One might argue that the number of embryos transferred does not matter when assessing the cumulative live birth rate. However, in the study by Goldman, all embryos from an oocyte retrieval were transferred when calculating the cumulative live birth rate, while in the present study, we had limited the time to embryo transfers performed within 1 year after oocyte retrieval. Thus, some embryos were still left in the freezer, which might increase the cumulative live birth rate to a small extent.

The number of cycles without embryo transfer, calculated per started fresh cycle, 16% (**Table 2**), is in line with current Q-IVF data (20) and also with United States data from the Centers for Disease Control and Prevention’s ART Report (33).

The association between BMI and maternal and perinatal outcomes after IVF has not been widely studied. A few, small Chinese studies found an increased risk of adverse maternal and perinatal outcomes in obese women compared with normal-weight women (17, 34, 35). However, BMI classes are defined differently in China and, thus, are less relevant for a non-Asian population (36). A large study in the United States in more than 60,000 singletons found that preterm birth significantly increased in obese women compared with normal-weight women (19).

The present study did not show an increased risk of major birth defects in children born to overweight and obese women compared with normal-weight women. A large Swedish population-based study of over 1.2 million singletons showed a higher rate of any major birth defects in overweight and obese women (13), and the difference is probably due to the inclusion of a lower number of singletons in the present study.

The mechanism behind how obesity affects the reproductive system is not well known. Different explanations have been suggested, most importantly that obese women have an increased rate of inflammatory and metabolic markers such as adipokines (37). Alterations in adipokine levels have been suggested to have a role in reproduction (38). The high grade of inflammation may affect both the endometrium (39) and oocyte quality (40). Bellver et al. found that obese oocyte recipients had lower live birth rates than normal-weight oocyte recipients (39), and Cardozo et al. reported decreased live birth rates in oocyte recipients of donors with increased BMI, suggesting that obesity may have an impact on the oocyte (40). During pregnancy, increased insulin resistance, alterations in placental structure and function characterized by increased inflammation, and epigenetic changes in offspring are some of the mechanisms that contribute to adverse consequences for the mother and child (41). Other suggested mechanisms include the endometrial microbiome system. Recently, an increasing interest is noticed in the balance between the endometrial microbiome and the immunological system at the endometrial level, both affecting endometrial receptivity, as recently reviewed by D’lppolito and coworkers (42). Studies have found a lower implantation rate in women with a non-*Lactobacillus*-dominated microbiome compared with women with a *Lactobacillus*-dominated microbiome and also that an aberrant distribution of endometrial immune molecules can be associated with poor obstetric outcomes, including miscarriage, PTB, preeclampsia, and intrauterine growth retardation. However, understanding of the microbiome system in relation to reproduction is still in its infancy.

It is well known that obesity is associated with a higher risk of recurrent pregnancy loss compared to normal-weight women, both after spontaneous conception (43) and after assisted reproduction (4, 8, 31). In a recent systematic review and meta-analysis, pooled data from 25 studies suggested that BMI in women with a history of recurrent pregnancy loss is significantly higher than BMI in controls (43). Although the exact mechanism is unknown, increased adiposity has been shown to disrupt the hypothalamic–pituitary–ovarian axis and steroidogenic activity in the ovary, through decreased insulin sensitivity, leading to metabolic syndrome and increased inflammation.

Hypertensive disorders of pregnancy, particularly preeclampsia/eclampsia, are a major cause of severe maternal and newborn morbidity and mortality. An increasing rate of preeclampsia has been linked to the parallel increase in maternal obesity (44, 45). While the cause of preeclampsia is still debated, the placenta seems to play a central role in its pathogenesis (46). A major theory is that improper placental vascularization results in placental ischemia, the release of vasoactive factors, and subsequent increased systemic inflammation, endothelial dysfunction, and hypertension. Novel evidence of normal and abnormal placental development is growing (47, 48). Obesity is a state of chronic, low-grade inflammation, and although the specific mechanisms whereby obesity increases the risk of preeclampsia are unclear, immune mechanisms and inflammation have been discussed (49–51). Recently, there has been increased interest in novel biomarkers for the prediction of preeclampsia (52).

Due to the decreased live birth rate and increased maternal and perinatal outcomes in obese women, several fertility clinics, especially those publicly funded, have set BMI limits that women must meet to be accepted for IVF treatment (53). In contrast, the view of the American Society for Reproductive Medicine is that obesity should not be the only reason for denying fertility treatment (54). The results from the present study can be used by clinicians in preconception counseling of obese women prior to IVF and possible referral for weight reduction before IVF for motivated obese women. However, female age seems to have a greater impact on fertility than high BMI especially for women above the age of 35 years (30). One might also argue, according to this study, that live birth rates are acceptable up to a BMI of 35 kg/m² or even some higher, but adverse maternal and perinatal outcomes increase substantially even at lower BMI levels.

Whether weight reduction prior to fertility treatment improves reproductive, maternal, and perinatal outcomes has not been thoroughly investigated. Randomized controlled trials have not been able to show improved live birth rate or cumulative live birth rate after weight reduction in obese women prior to fertility treatment (55–58), and the women had regained prestudy weight after 2 years (57). These trials showed no difference in maternal and perinatal outcomes; however, they were not powered to investigate the effect of weight reduction on these outcomes (56, 59). Trials are scarce on the effect of pre-pregnancy behavioral weight loss interventions to improve maternal and perinatal outcomes in women with obesity in the general population. A recent trial found that although women in the intervention group lost weight before pregnancy, they gained more weight in late pregnancy. Except for a lower early pregnancy loss in the intervention group, maternal and perinatal outcomes were similar (60). A systematic review and meta-analysis of women who became pregnant after bariatric surgery showed reduced risks of gestational diabetes, hypertensive disorders, and large-for-gestational-age infants. Yet, it also showed an increased risk of small for gestational age and preterm birth in these women compared with women with similar prebariatric BMI (61). Further research, although challenging, is needed to determine whether weight reduction prior to IVF can reduce the adverse outcomes in obese women.

## Conclusion

In conclusion, in a large and complete national cohort of women undergoing IVF, we demonstrate a dose-dependent decrease in live birth rates and a substantial increase in maternal and perinatal complications with increasing BMI, giving new insights to the further consequences of maternal overweight and obesity in IVF. Strategies to improve this situation are warranted.

## Data availability statement

The data analyzed in this study is subject to the following licenses/restrictions: The data underlying this article cannot be shared publicly owing to restrictions by law. The source of the data for this study was national register information given for this specific study. The use of microdata from the national registers follows the rules and regulations of the Swedish General Data Protection Regulation (GDPR) agency. Due to data protection and privacy legislations no data are available. Further information on the registers is available from the Swedish National Board of Health and Welfare (https://www.socialstyrelsen.se/en/statistics-and-data/registers/). Requests to access these datasets should be directed to See above.

## Ethics statement

The studies involving humans were approved by the National Research Ethics Committee in Stockholm (DNR 2020-07126). The studies were conducted in accordance with the local legislation and institutional requirements. Written informed consent for participation was not required from the participants or the participants’ legal guardians/next of kin in accordance with the national legislation and institutional requirements.

## Author contributions

CB formed the study concept. All authors contributed to the design of the study and wrote the statistical analysis plan. KK did the statistical analysis. LK and CB acquired the data. LK, AT-K, and CB obtained the funding. LK, KK, AT-K, U-BW, and CB interpreted the data. LK wrote the first draft of the manuscript, which was then critically reviewed and revised by the other co-authors. All authors approved the final version of the manuscript for submission. LK and CB are guarantors. The corresponding author attests that all listed authors meet authorship and that no others meeting the criteria have been omitted.
