# Supplementary material for: The association between body mass index and live birth and maternal and perinatal outcomes after in-vitro fertilization: a national cohort study
Source: Front Endocrinol (Lausanne). 2023 Sep 11;14:1239702. doi: 10.3389/fendo.2023.1239702 (PMC10520462; doi:10.3389/fendo.2023.1239702)
Supplement: Supplementary file 1 [file Table_1.docx]

Supplementary Material

**The association between body mass index and live birth and maternal and perinatal outcomes after in vitro fertilisation: A national cohort study**

Linda Kluge ^1,2*^, Karin Källén^3^, Ann Thurin-Kjellberg^1,2^, Ulla-Britt Wennerholm^1,4^, Christina Bergh ^1,2^

*^1^Department of Obstetrics and Gynaecology, Institute of Clinical Science, Sahlgrenska Academy, Gothenburg University, Gothenburg, Sweden.*

*^2^Reproductive Medicine, Sahlgrenska University Hospital, Gothenburg, Sweden.*

*^3^Department of Reproduction Epidemiology, Tornblad Institute, Institute of Clinical Science, Lund University, Lund, Sweden.*

*^4^Region Västra Götaland, Sahlgrenska University Hospital, Department of Obstetrics and Gynaecology, Gothenburg, Sweden.*

*** Correspondence:** Corresponding Author: linda.kluge@vgregion.se

# Supplementary Table S1. Year of started fresh cycle* stratified by BMI class

*Started fresh cycle = a fresh cycle where at least one dose of gonadotrophins was administered

BMI = body mass index, n = number

|  | **BMI class** | | | | | | | | | | | |  |  |  |
| --- | --- | --- | --- | --- | --- | --- | --- | --- | --- | --- | --- | --- | --- | --- | --- |
| Year of started fresh cycle | <18.5 | | 18.5–24.9 | | 25–29.9 | | 30–34.9 | | 35–39.9 | | ≥40 | | BMI not known | | Total |
|  | n | (%) | n | (%) | n | (%) | n | (%) | n | (%) | n | (%) | n | (%) | n |
| 2007 | 142 | (1.4) | 4022 | (39.9) | 1473 | (14.6) | 453 | (4.5) | 102 | (1.0) | 5 | (0.0) | 3891 | (38.6) | 10 088 |
| 2008 | 201 | (1.8) | 4893 | (44.8) | 1739 | (15.9) | 522 | (4.8) | 73 | (0.7) | 9 | (0.1) | 3496 | (32.0) | 10 933 |
| 2009 | 202 | (1.7) | 5487 | (47.4) | 2012 | (17.4) | 669 | (5.8) | 100 | (0.9) | 12 | (0.1) | 3095 | (26.7) | 11 577 |
| 2010 | 248 | (2.1) | 6312 | (54.6) | 2508 | (21.7) | 781 | (6.8) | 150 | (1.3) | 7 | (0.1) | 1556 | (13.5) | 11 562 |
| 2011 | 268 | (2.3) | 6828 | (57.6) | 2680 | (22.6) | 875 | (7.4) | 120 | (1.0) | 8 | (0.1) | 1074 | (9.1) | 11 853 |
| 2012 | 242 | (2.1) | 6684 | (57.1) | 2700 | (23.1) | 923 | (7.9) | 119 | (1.0) | 9 | (0.1) | 1024 | (8.8) | 11 701 |
| 2013 | 261 | (2.3) | 6252 | (54.6) | 2587 | (22.6) | 868 | (7.6) | 110 | (1.0) | 3 | (0.0) | 1373 | (12.0) | 11 454 |
| 2014 | 242 | (2.0) | 6357 | (53.8) | 2516 | (21.3) | 910 | (7.7) | 129 | (1.1) | 4 | (0.0) | 1666 | (14.1) | 11 824 |
| 2015 | 263 | (2.2) | 6457 | (53.8) | 2719 | (22.6) | 941 | (7.8) | 97 | (0.8) | 2 | (0.0) | 1528 | (12.7) | 12 007 |
| 2016 | 261 | (2.1) | 6614 | (53.8) | 2830 | (23.0) | 983 | (8.0) | 83 | (0.7) | 1 | (0.0) | 1519 | (12.4) | 12 291 |
| 2017 | 251 | (2.1) | 6460 | (53.9) | 2839 | (23.7) | 1069 | (8.9) | 94 | (0.8) | 4 | (0.0) | 1269 | (10.6) | 11 986 |
| 2018 | 257 | (2.2) | 5937 | (51.5) | 2828 | (24.5) | 986 | (8.5) | 98 | (0.8) | 0 | (0.0) | 1432 | (12.4) | 11 538 |
| 2019 | 246 | (2.0) | 6221 | (51.7) | 3082 | (25.6) | 1056 | (8.8) | 122 | (1.0) | 2 | (0.0) | 1304 | (10.8) | 12 033 |
| Total | 3084 | (2.0) | 78 524 | (52.1) | 32 513 | (21.6) | 11 036 | (7.3) | 1397 | (0.9) | 66 | (0.0) | 24 227 | (16.1) | 150 847 |

**Supplementary Table S2. Characteristics of subpopulation 1 stratified by availability** **of BMI information**

|  |  | **BMI known** | | | | | **BMI missing** | | | |
| --- | --- | --- | --- | --- | --- | --- | --- | --- | --- | --- |
|  |  | N=126 620 | | | | | N=24 227 | | | |
|  |  | n | | | (%) | | n | | (%) | |
| Woman's age (years) | |  | | |  | |  | |  | |
|  | <30 | 22 954 | | | (18.1) | | 3332 | | (13.8) | |
|  | 30–34 | 42 562 | | | (33.6) | | 7719 | | (31.9) | |
|  | 35–37 | 28 578 | | | (22.6) | | 5609 | | (23.2) | |
|  | 38–39 | 18 236 | | | (14.4) | | 3817 | | (15.8) | |
|  | ≥40 | 14 290 | | | (11.3) | | 3750 | | (15.5) | |
| Parity (previous children) | |  | |  | |  | |  | |  |
|  | No | 97 715 | | | (77.2) | | 18 044 | | (74.5) | |
|  | Yes | 28 905 | | | (22.8) | | 6183 | | (25.5) | |
| Country of birth | |  | | |  | |  | |  | |
|  | Sweden | 97 017 | | | (76.6) | | 18 433 | | (76.1) | |
|  | Other European | 11 596 | | | (9.2) | | 2252 | | (9.3) | |
|  | Outside Europe | 18 007 | | | (14.2) | | 3542 | | (14.6) | |
| Cause of infertility | |  | | |  | |  | |  | |
|  | Male factor | 32 846 | | | (25.9) | | 6586 | | (27.2) | |
|  | Tubal factor | 8141 | | | (6.4) | | 1258 | | (5.2) | |
|  | Endometriosis | 7355 | | | (5.8) | | 1239 | | (5.1) | |
|  | Female factor not specified | 28 838 | | | (22.8) | | 3677 | | (15.2) | |
|  | PCOS | 6800 | | | (5.4) | | 865 | | (3.6) | |
|  | Anovulation | 13 764 | | | (10.9) | | 3544 | | (14.6) | |
|  | Unexplained or not known | 41 760 | | | (33.0) | | 9072 | | (37.4) | |
| Educational level (years) | |  | | |  | |  | |  | |
|  | ≤9 | 10 132 | | | (8.0) | | 1858 | | (7.7) | |
|  | 10–12 | 33 295 | | | (26.3) | | 6052 | | (25.0) | |
|  | ≥13 | 82 327 | | | (65.0) | | 16 083 | | (66.4) | |
|  | Not known | 866 | | | (0.7) | | 234 | | (1.0) | |
| Previous failed fresh cycles | | |  | |  | |  | |  | |
|  | 0 | 67 534 | | | (53.3) | | 13 375 | | (55.2) | |
|  | 1 | 30 778 | | | (24.3) | | 5473 | | (22.6) | |
|  | 2 | 15 176 | | | (12.0) | | 2687 | | (11.1) | |
|  | ≥3 | 13 132 | | | (10.4) | | 2692 | | (11.1) | |
| Previous children with IVF | |  | | |  | |  | |  | |
|  | No | 112 331 | | | (88.7) | | 21 546 | | (88.9) | |
|  | Yes | 14 289 | | | (11.3) | | 2681 | | (11.1) | |
| Year of started fresh cycle* | |  | | |  | |  | |  | |
|  | 2007–2011 | 42 901 | | | (33.9) | | 13 112 | | (54.1) | |
|  | 2012–2015 | 41 395 | | | (32.7) | | 5591 | | (23.1) | |
|  | 2016–2019 | 42 324 | | | (33.4) | | 5524 | | (22.8) | |
| Fertilisation method | |  | | |  | |  | |  | |
|  | IVF | 50 530 | | | (39.9) | | 8949 | | (36.9) | |
|  | ICSI | 47 705 | | | (37.7) | | 9314 | | (38.4) | |
| Retrieved oocytes | |  | | |  | |  | |  | |
|  | <5 | 21 494 | | | (17.0) | | 4240 | | (17.5) | |
|  | 5-9 | 46 045 | | | (36.4) | | 8313 | | (34.3) | |
|  | 10-19 | 45 954 | | | (36.3) | | 8102 | | (33.4) | |
|  | ≥20 | 6685 | | | (5.3) | | 1142 | | (4.7) | |
| Presence and type of fresh ET | |  | | |  | |  | |  | |
|  | No fresh ET | 25 287 | | | (20.0) | | 5731 | | (23.7) | |
|  | Fresh SET | 79 557 | | | (62.8) | | 13 790 | | (56.9) | |
|  | Fresh DET | 21 776 | | | (17.2) | | 4706 | | (19.4) | |
| Number of frozen ETs  per oocyte aspiration | |  | | | | |  | |  | |
|  | No frozen ET | 95 918 | | | (75.8) | | 19 332 | | (79.8) | |
|  | 1–2 frozen ETs | 27 627 | | | (21.8) | | 4530 | | (18.7) | |
|  | 3–4 frozen ETs | 2873 | | | (2.3) | | 343 | | (1.4) | |
|  | 5 frozen ETs | 202 | | | (0.2) | | 22 | | (0.1) | |

*Started fresh cycle = a fresh cycle where at least one dose of gonadotrophins was administered

DET = double embryo transfer, ET = embryo transfer, ICSI = intracytoplasmic sperm injection,

IVF = in vitro fertilisation, PCOS = polycystic ovary syndrome, SET = single embryo transfer

| **Supplementary Table S3. Live birth per started fresh cycle, cumulative live birth per started fresh cycle*, live birth per fresh embryo transfer stratified by availability of BMI information** | | | | | | | | | |
| --- | --- | --- | --- | --- | --- | --- | --- | --- | --- |
|  | **Live birth per started fresh cycle*** | | **Cumulative live birth per**  **started fresh cycle** | | **Started fresh cycles** |  | **Live birth per fresh ET** | | **Fresh ET** |
| BMI | n | (%) | n | (%) | n |  | n | (%) | n |
| BMI known | 27 809 | (22.0) | 39 500 | (31.2) | 126 620 |  | 27 798 | (27.4) | 101 333 |
| BMI not known | 4978 | (20.5) | 6846 | (28.3) | 24 227 |  | 4975 | (26.9) | 18 496 |

*started fresh cycle = a fresh cycle where at least one dose of gonadotrophins was administered

BMI = body mass index, ET = embryo transfer, n = number

**Supplementary Table S4. Miscarriage among clinical pregnancies^*^ after first embryo transfer in relation to BMI class**

|  | **Miscarriages after first ET** | | **Clinical Pregnancies after first ET** | **Risk Ratio** | |  | **Adjusted Risk Ratio^†^** | | **p-value** |
| --- | --- | --- | --- | --- | --- | --- | --- | --- | --- |
| BMI kg/m² | n | (%) | n | RR | 95% CI |  | ARR | 95% CI |  |
| <18.5 | 154 | (15.4) | 1002 | 0.91 | 0.78 to1.05 |  | 0.95 | 0.82 to 1.10 | 0.469 |
| 18.5–24.9 | 4474 | (17.1) | 26 146 | 1.0 | reference |  | 1.0 | reference |  |
| 25–29.9 | 1949 | (18.9) | 10 339 | 1.13 | 1.08 to 1.18 |  | 1.10 | 1.05 to 1.15 | <0.000 |
| 30–34.9 | 726 | (21.1) | 3443 | 1.25 | 1.17 to 1.34 |  | 1.21 | 1.13 to 1.29 | <0.000 |
| ≥35 | 104 | (25.9) | 401 | 1.54 | 1.31 to 1.82 |  | 1.36 | 1.16 to 1.60 | <0.000 |

^*^Clinical pregnancy = ultrasonographic visualisation of one or more gestational sacs.

**^†^**Adjusted for year of treatment (continuous), maternal age (continuous), country of birth (Sweden/Other European/Outside Europe), educational level (ordinal), type of IVF-treatment (IVF/ICSI), number of previous failed fresh cycles (continuous), number of previous IVF children (continuous).

ARR = adjusted risk ratio, CI = confidence interval, ET = embryo transfer, n = number, RR = risk ratio

**Supplementary Table S5. Multifetal pregnancies per delivery after first embryo transfer in relation to BMI class**

| BMI kg/m² | **Multifetal pregnancies**  **after first ET** | | **Deliveries after**  **first ET** | **Risk Ratio** | |  | **Adjusted Risk Ratio*** | |  | **Adjusted Risk Ratio†** | | **p-value** |
| --- | --- | --- | --- | --- | --- | --- | --- | --- | --- | --- | --- | --- |
|  | n | (%) | n | RR | 95% CI |  | ARR | 95% CI |  | ARR | 95% CI |  |
| <18.5 | 34 | (4.5) | 756 | 1.03 | 0.74 to 1.45 |  | 1.05 | 0.75 to 1.46 |  | 1.10 | 0.81 to 1.49 | 0.544 |
| 18.5–24.9 | 836 | (4.3) | 19 313 | 1.0 | reference |  | 1.0 | reference |  | 1.0 | reference |  |
| 25–29.9 | 310 | (4.2) | 7364 | 0.98 | 0.87 to 1.15 |  | 1.02 | 0.90 to1.15 |  | 0.98 | 0.88 to 1.11 | 0.807 |
| 30–34.9 | 125 | (5.4) | 2320 | 1.26 | 1.05 to 1.50 |  | 1.34 | 1.12 to 1.60 |  | 1.33 | 1.12 to 1.58 | 0.001 |
| ≥35 | 16 | (6.3) | 252 | 1.46 | 0.90 to 2.35 |  | 1.34 | 0.82 to 2.21 |  | 1.42 | 0.90 to 2.26 | 0.133 |

*Adjusted for year of treatment (continuous), maternal age (continuous), country of birth (Sweden/other European/Outside Europe), educational level (ordinal), type of IVF-treatment (IVF/ICSI), number of previous failed fresh cycles (continuous), number of previous IVF children (continuous).

^†^Adjusted for all factors listed under*, and in addition for number of embryos transferred at first ET.

ARR adjusted risk ratio, BMI = body mass index, CI = confidence interval, ET = embryo transfer, n = number, RR = risk ratio

| **Supplementary Table S6. Year of IVF among treatments leading to delivery stratified by BMI class** | | | | | | | | | | | | | | | |
| --- | --- | --- | --- | --- | --- | --- | --- | --- | --- | --- | --- | --- | --- | --- | --- |
|  | **BMI class** | | | | | | | | | | | |  |  |  |
| IVF (year) | <18.5 | | 18.5–24.9 | | 25–29.9 | | 30–34.9 | | 35–39.9 | | ≥40 | | BMI not known | | Total |
|  | n | (%) | n | (%) | n | (%) | n | (%) | n | (%) | n | (%) | n | (%) | n |
| 2002 | 20 | (1.2) | 894 | (53.2) | 377 | (22.5) | 129 | (7.7) | 30 | (1.8) | 6 | (0.4) | 223 | (13.3) | 1679 |
| 2003 | 18 | (0.9) | 1068 | (52.5) | 473 | (23.3) | 166 | (8.2) | 50 | (2.5) | 9 | (0.4) | 250 | (12.3) | 2034 |
| 2004 | 21 | (0.9) | 1220 | (54.2) | 521 | (23.1) | 160 | (7.1) | 48 | (2.1) | 7 | (0.3) | 275 | (12.2) | 2252 |
| 2005 | 20 | (0.8) | 1334 | (54.6) | 592 | (24.2) | 171 | (7.0) | 30 | (1.2) | 8 | (0.3) | 290 | (11.9) | 2445 |
| 2006 | 43 | (1.5) | 1537 | (53.1) | 631 | (21.8) | 239 | (8.3) | 53 | (1.8) | 9 | (0.3) | 380 | (13.1) | 2892 |
| 2007 | 50 | (1.9) | 1653 | (63.5) | 595 | (22.9) | 169 | (6.5) | 24 | (0.9) | 5 | (0.2) | 107 | (4.1) | 2603 |
| 2008 | 70 | (2.5) | 1885 | (66.5) | 635 | (22.4) | 150 | (5.3) | 23 | (0.8) | 4 | (0.1) | 66 | (2.3) | 2833 |
| 2009 | 71 | (2.4) | 1939 | (64.5) | 705 | (23.5) | 203 | (6.8) | 35 | (1.2) | 0 | (0.0) | 51 | (1.7) | 3004 |
| 2010 | 65 | (1.9) | 2228 | (65.5) | 798 | (23.5) | 242 | (7.1) | 33 | (1.0) | 1 | (0.0) | 32 | (0.9) | 3399 |
| 2011 | 98 | (2.9) | 2258 | (66.5) | 774 | (22.8) | 221 | (6.5) | 25 | (0.7) | 1 | (0.0) | 21 | (0.6) | 3398 |
| 2012 | 85 | (2.6) | 2192 | (65.8) | 784 | (23.5) | 232 | (7.0) | 24 | (0.7) | 2 | (0.1) | 11 | (0.3) | 3330 |
| 2013 | 90 | (2.6) | 2303 | (66.5) | 813 | (23.5) | 213 | (6.2) | 25 | (0.7) | 1 | (0.0) | 18 | (0.5) | 3463 |
| 2014 | 92 | (2.6) | 2364 | (66.5) | 812 | (22.8) | 241 | (6.8) | 24 | (0.7) | 2 | (0.1) | 21 | (0.6) | 3556 |
| 2015 | 100 | (2.5) | 2675 | (65.8) | 953 | (23.4) | 274 | (6.7) | 32 | (0.8) | 2 | (0.0) | 28 | (0.7) | 4064 |
| 2016 | 95 | (2.2) | 2786 | (65.5) | 988 | (23.2) | 312 | (7.3) | 31 | (0.7) | 2 | (0.0) | 42 | (1.0) | 4256 |
| 2017 | 100 | (2.2) | 2875 | (64.0) | 1091 | (24.3) | 370 | (8.2) | 26 | (0.6) | 0 | (0.0) | 31 | (0.7) | 4493 |
| 2018 | 120 | (2.7) | 2819 | (63.6) | 1076 | (24.3) | 357 | (8.1) | 26 | (0.6) | 0 | (0.0) | 32 | (0.7) | 4430 |
| 2019 | 106 | (2.2) | 2937 | (61.1) | 1300 | (27.0) | 403 | (8.4) | 32 | (0.7) | 0 | (0.0) | 28 | (0.6) | 4806 |
| 2020 | 25 | (2.2) | 734 | (63.4) | 293 | (25.3) | 100 | (8.6) | 4 | (0.3) | 0 | (0.0) | 2 | (0.2) | 1158 |
| Total | 1289 | (2.1) | 37 701 | (62.7) | 14 211 | (23.6) | 4352 | (7.2) | 575 | (1.0) | 59 | (0.1) | 1908 | (3.2) | 60 095 |
| BMI = body mass index, n = number | | | | | | | | | | | | | | | |

**
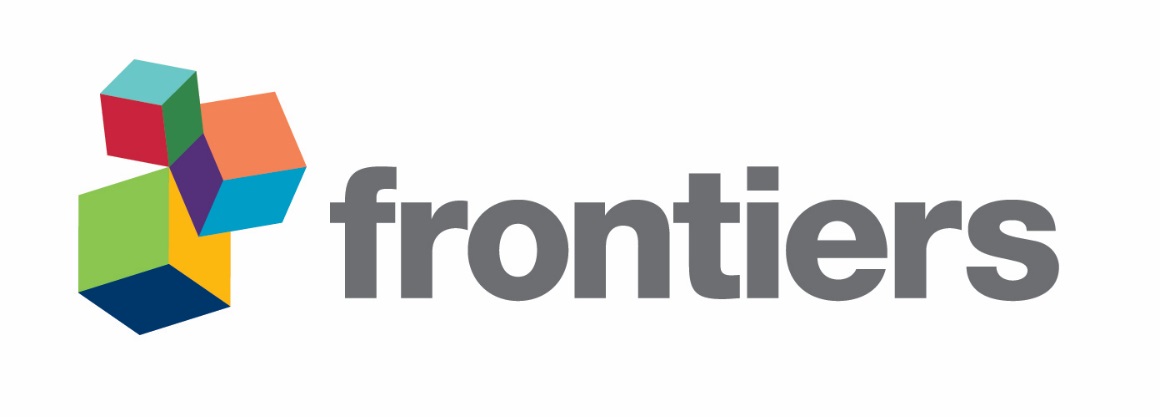
**
